# Facile Halogenation of Antimicrobial Peptides As Demonstrated by Producing Bromotryptophan-Labeled Nisin Variants with Enhanced Antimicrobial Activity

**DOI:** 10.1021/acs.jnatprod.4c00118

**Published:** 2024-06-18

**Authors:** Longcheng Guo, Oscar P. Kuipers, Jaap Broos

**Affiliations:** Department of Molecular Genetics, Groningen Biomolecular Sciences and Biotechnology Institute, University of Groningen, Groningen 9747, AG, The Netherlands

## Abstract

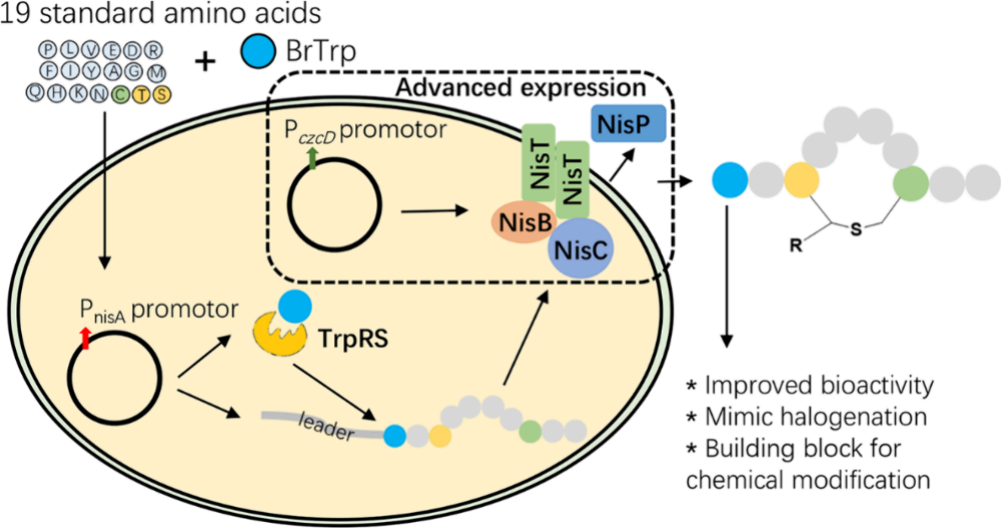

Antimicrobial peptides (AMPs) have raised significant interest, forming a potential new class of antibiotics in the fight against multi-drug-resistant bacteria. Various AMPs are ribosomally synthesized and post-translationally modified peptides (RiPPs). One post-translational modification found in AMPs is the halogenation of Trp residues. This modification has, for example, been shown to be critical for the activity of the potent AMP NAI-107 from *Actinoallomurus*. Due to the importance of organohalogens, establishing methods for facile and selective halogen atom installation into AMPs is highly desirable. In this study, we introduce an expression system utilizing the food-grade strain *Lactococcus lactis*, facilitating the efficient incorporation of bromo-Trp (BrTrp) into (modified) peptides, exemplified by the lantibiotic nisin with a single Trp residue or analogue incorporated at position 1. This provides an alternative to the challenges posed by halogenase enzymes, such as poor substrate selectivity. Our method yields expression levels comparable to that of wild-type nisin, while BrTrp incorporation does not interfere with the post-translational modifications of nisin (dehydration and cyclization). One brominated nisin variant exhibits a 2-fold improvement in antimicrobial activity against two tested pathogens, including a WHO priority pathogen, while maintaining the same lipid II binding and bactericidal activity as wild-type nisin. The work presented here demonstrates the potential of this methodology for peptide halogenation, offering a new avenue for the development of diverse antimicrobial products labeled with BrTrp.

RiPPs are ribosomally synthesized and post-translationally modified peptides that form a major class of natural products.^1,2^ Lanthipeptides, forming the most extensively studied RiPP class, are characterized by cross-linked thioether structures called lanthionine (Lan) and methyllanthionine (MeLan).^3,4^ Nisin (Figure 1A), one of the best characterized lanthipeptides, has been widely used as a food preservative for many years due to its potent antimicrobial activity and excellent safety profile.^5^ Additionally, nisin has demonstrated therapeutic potential against various Gram-positive antibiotic-resistant organisms, including vancomycin-resistant *Enterococcus* and methicillin-resistant *Staphylococcus aureus.*(5) However, the peptidic nature of nisin limits its application due to susceptibility to proteolytic degradation, toxicity and/or immunogenicity concerns, and poor pharmacokinetics.^6^ Nisin’s gene-encoded synthesis and relatively simple biosynthesis pathway make it an excellent candidate for further engineering, expanding the diversity of the antimicrobial activity arsenal. To this end, a variety of engineering strategies have been employed, including point mutations, modular shuffling, introduction of diverse heterologous post-translational modifications, chemical modifications, and incorporation of noncanonical amino acid (ncAA).^7−9^

**Figure 1:**
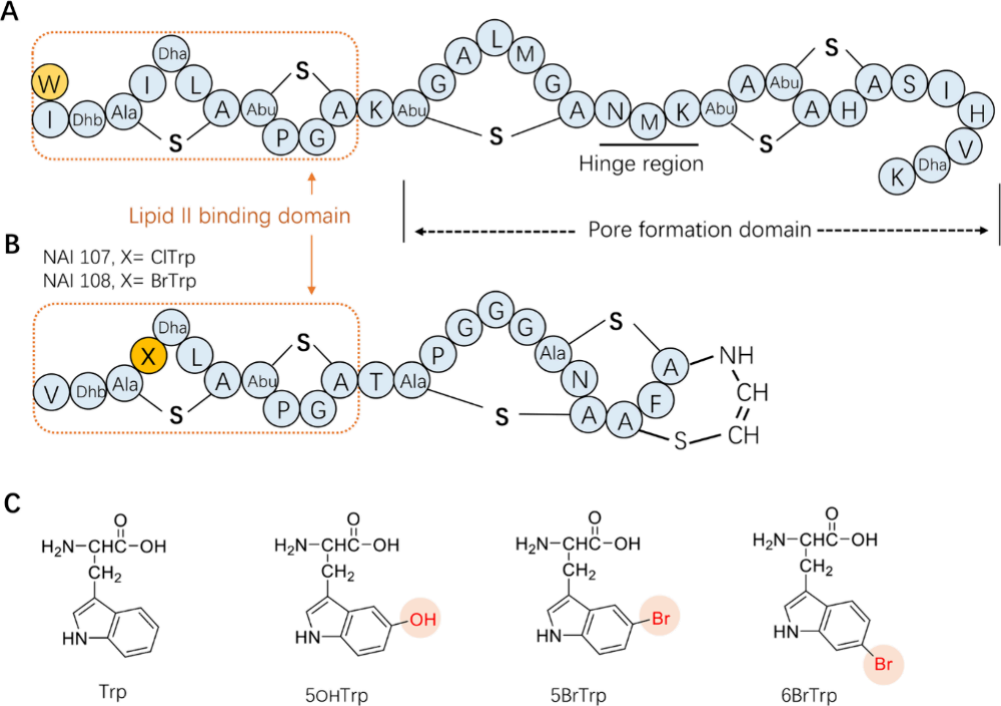
Structures of nisin, lantibiotics NAI-107/108, and Trp analogues used in this study. (A) Structure of nisin A and its variant nisin(I1W). Dha, dehydroalanine; Dhb, dehydrobutyrine; Ala-S-A, lanthionine; Abu-S-A, methyllanthionine; the functional domains, lipid II binding site, pore formation domain, and hinge region are indicated. (B) Structures of lantibiotic NAI-107/108 identified from *Actinoallomurus*. The Trp modification is highlighted in yellow. (C) Structures of Trp and its analogues used in this study: 5OHTrp, 5-hydroxy-Trp; 5BrTrp, 5-bromo-Trp; 6BrTrp, 6-bromo-Trp.

Halogenation is an important strategy for tuning and enhancing the bioavailability and bioactivity of molecules, gaining widespread attention from both academia and industry.^10^ Due to its beneficial properties, halogenation is extensively employed by agrochemical and pharmaceutical industries, with approximately 20% of small-molecule drugs and 30% of agrochemical compounds being halogenated.^11^ Additionally, introducing halogens into molecules creates sites for specific functionalization through cross-coupling chemistries.^12^ However, traditional synthetic methods for halogenation often involve harsh conditions, lack regioselectivity, employ harmful reagents, or generate undesirable byproducts.^13^ Although halogenase enzymes show potential, their use is often limited by poor efficiency, selectivity issues, and unsuitability for complex substrates.^14^ Hence, there is a need for reliable, straightforward, and cleaner halogenation methods.

Usually, halogenation happens on aromatic amino acids.^13^ Nisin A does not contain the residues Phe, Tyr, and Trp (Figure 1A), which are often crucial for the biological activity of many peptides and proteins.^15^ A recent study reported that introducing a Trp or Phe residue at position 1 of nisin increases the antimicrobial activity against an *L. lactis* strain.^16^ Trp has also been introduced at a few other residue positions, and the antimicrobial activity of these single-Trp nisin constructs was somewhat lower compared to wild-type nisin.^17,18^ The *L. lactis* Trp auxotroph strain PA1002 has been employed in a forced feeding approach to incorporate Trp analogues in these single-Trp nisin constructs, and to prevent the labeling of the nisin modification enzymes with the Trp analogue, a cross-expression system was developed.^18^ In the first phase, the expression of the nisin modification machinery was initiated in a rich medium and subsequently shifted to a new synthetic medium lacking Trp but containing a Trp analogue for the production of nisin (Figure 2B). The Trp analogues 5-fluoro-Trp, 5-hydroxy-Trp, and 5-methyl-Trp were successfully incorporated into four single-Trp nisin constructs (positions 1, 4, 17, and 32). The incorporation efficiency of Trp analogues varied from 69% to 97%.^18^ In this work, we further explore the potential of Trp analogues in antimicrobial peptides. Halogenation of Trp provides many benefits over other types of modifications.^19,20^ Halogens can modulate ligand–receptor interactions through their electron-withdrawing features, polarizability, and steric effects, controlling the degradability, lipophilicity, membrane permeabilization, and catabolic stability of pharmaceuticals.^21,22^ For example, NAI-107, one of the most potent lantibiotics reported so far,^23^ belongs to the same class as nisin, with which it shares the two N-terminal lanthionine rings (Figure 1B). Notably, NAI-107 features a distinctive 5-chloroTrp residue, and the chlorination is a post-translational modification proven to contribute to its high antibacterial activity.^24^ Interestingly, substituting the chlorine with bromine, named NAI-108 (Figure 1B), can further enhance the antibacterial potency of NAI-107.^25^

**Figure 2:**
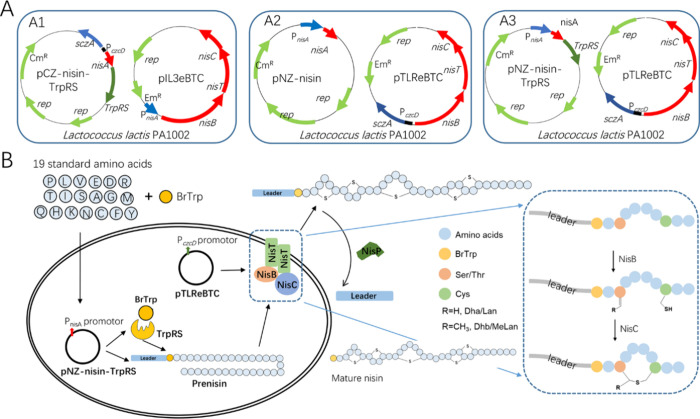
Schematic overview of the cross-expression systems used to incorporate ncAA into nisin. (A1) Cross-expression system (pCZ-nisin system) has been developed before for the incorporation of 5-fluoro-Trp, 5-hydroxy-Trp, and 5-methyl-Trp into nisin.^18^ (A2) A cross-expression system (pNZ-nisin) developed for incorporation of Met analogues.^29^ (A3) A cross-expression system developed in this study for the incorporation of BrTrp in nisin. *SczA*, encoding the repressor of P_*czcD*_; P_*czcD*_, a zinc inducible promoter; *nisA*, encoding NisA; P_*nisA*_, a nisin-inducible promoter; *trpRS*, encoding tryptophanyl-tRNA synthetase from *L. lactis*; *nisB*, *nisT*, and *nisC*, encoding nisin modification machinery NisBTC; *rep*, encoding plasmid replication proteins; *Cm*^*R*^, chloramphenicol resistance gene; and *Em*^*R*^, erythromycin resistance gene. (B) Schematic overview of the force-feeding method used to incorporate a Trp analogue into nisin. The cross-expression system allows for differential timing of the expression of the lantibiotic and its modification enzymes. After the expression of NisBTC in the presence of 20 canonical amino acids, the washed cells are suspended in synthetic medium supplemented with 19 canonical amino acids and a Trp analogue and the nisin to induce expression of prenisin. The precursor nisin (prenisin) consists of a leader peptide and a core peptide that undergoes post-translational modifications catalyzed by the NisBTC (dotted box). Specifically, NisB dehydrates Ser and Thr to form dehydro residues, which can then be coupled to Cys to form (methyl)lanthionine rings catalyzed by NisC. Finally, the transporter NisT exports the modified prenisin outside the cells, where the protease NisP removes the leader peptide, releasing mature modified nisin.

Biosynthetic incorporation of halogenated Trp analogues in proteins has been demonstrated for mono-, di-, tri-, and tetrafluoroTrp analogues^26^ and monochlorinated Trp.^27^ 5BrTrp and 6BrTrp can be translated using the *L. lactis* Trp auxotroph strain PA1002,^27^ and orthogonal Trp synthethase/tRNA^Trp^ pairs have been developed for the incorporation of 5ChloroTrp and 5BrTrp in proteins using *E. coli* as host.^28^ No study has reported the incorporation of ChloroTrp or BrTrp in the ribosomally synthesized and post-translationally modified peptides (RiPPs).

Introducing halogenated Trp analogues in nisin or other RiPPs could become a route to enhance its antimicrobial activity and change its specificity, but explorative experiments using the developed cross-expression system^18^ indicated that brominated Trp analogues are not efficiently introduced during ribosomal biosynthesis. We note that of all halogenated Trp analogues introduced using *L. lactis* in non-post-translationally modified proteins, labeling with the bulky BrTrp was most challenging, as reflected in a lower protein yield and somewhat lower incorporation efficiencies. In this work, we redesigned the cross-expression system, making it suitable for the efficient incorporation of the brominated Trp analogues in nisin. Labeling a protein with 5-hydroxy-Trp^27^ was also found sensitive for expression conditions, e.g., overexpression of TrpRS, and this analogue was included to find optimal conditions for labeling a RiPP with a Trp analogue. The resulting nisin variants were purified and characterized, and their properties that were occasionally improved are reported herein.

## Results and Discussion

### An Expression System for Incorporating Bromo-Trp into Nisin

Nisin is a peptide synthesized through ribosomal processes and subjected to post-translational modifications. The synthesis of nisin (Figure 1A) involves the expression of *nisABTC* genes, responsible for the nisin modification machinery (NisBTC) and the production of prenisin, a modified core nisin with an N-terminal leader segment attached (Figure 2). The leader part serves the crucial role of guiding the core peptide through the NisBTC modification machinery. NisB dehydrates 3 Ser and 5 Thr residues in prenisin to dehydroalanines (Dha) and dehydrobutyrines (Dhbs), respectively, and NisC subsequently catalyzes the formation of 5 (methyl)lanthionine rings involving 5 Cys, 1 Dha, and 4 Dhb residues. After transport over the outer membrane by NisT, nisin is activated by NisP, which cleaves off the N-terminal leader (Figure 2B). The two plasmids used in the earlier developed cross-expression system for the incorporation of Trp analogues in nisin and transformed into *L. lactis* Trp auxotroph PA1002 strain are presented in Figure 2A1.^18^ Translation of the genes for the nisin modification machinery NisBTC, controlled by the P_*nisA*_ promoter (plasmid pil3eBTC), is initiated while PA1002 is growing in rich medium (Figure S1). After exchange of the growth medium to chemically defined medium (CDM), supplemented with a Trp analogue, translation of the single-Trp-containing nisin construct nisin(I1W) and native tryptophanyl-tRNA synthetase (TrpRS) is induced by adding 0.5 mM Zn^2+^ (plasmid pCZ-nisin(I1W)-TrpRS). In this work we refer to this system as the “pCZ-nisin system”, as the zinc promoter P_*czcD*_ is used to control the nisin expression. To enhance the translation of more bulky Trp analogues like BrTrp (Figure 1C), the impact of swapping the two promoters P_*nisA*_ and P_*czcD*_ was investigated (Figure 2A2). In this system, referred to as the pNZ-nisin system, nisin expression is controlled by the strong nisin promoter P_*nisA*_ (Figure 2A2). This system had been shown to improve the yield and incorporation efficiency for Met analogues.^29^ The impact of overexpression of TrpRS by P_*nisA*_ in the pNZ-nisin system was also investigated (Figure 2A3).

### Expression of Nisin(I1W) Labeled with a Trp Analogue

**Figure 3:**
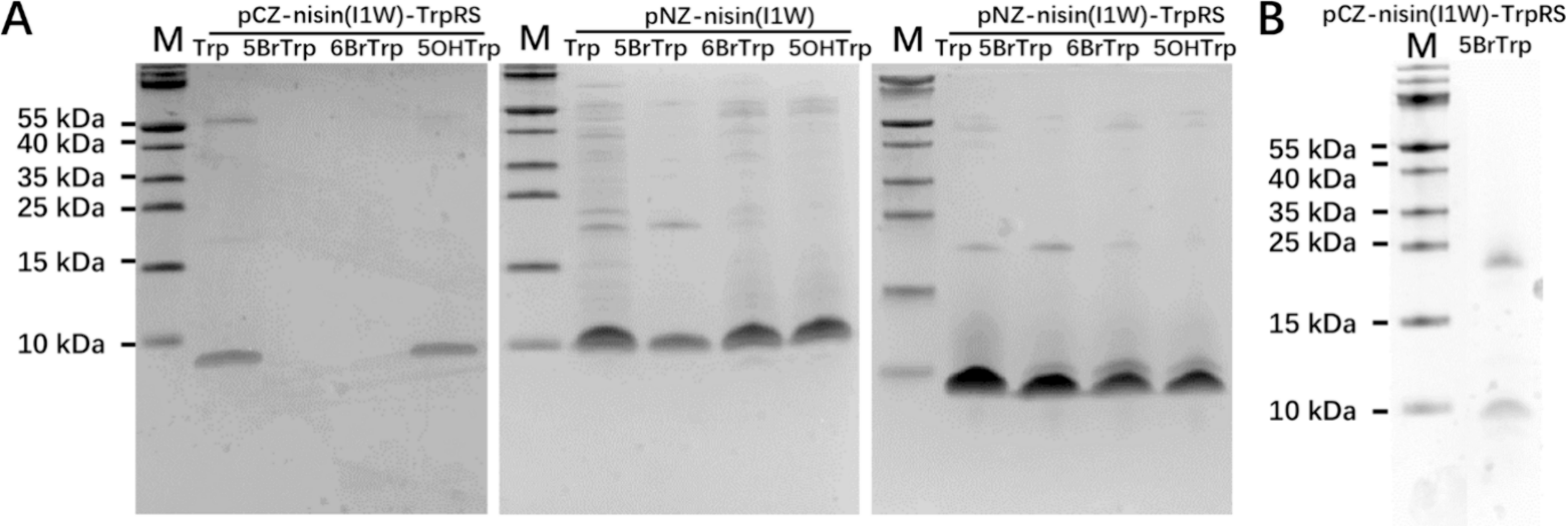
(A) Tricine-SDS-PAGE gel stained with Coomassie blue illustrating the incorporation of analogues using three cross-expression systems. Each well contains TCA-precipitated peptides from 1 mL of supernatant. (B) Tricine-SDS-PAGE gel analysis of 10 times concentrated supernatant using the pCZ-nisin(I1W)-TrpRS system for 5BrTrp incorporation. Ten mL of supernatant was concentrated to 1 mL, followed by TCA precipitation. M: protein marker (Biolabs); 5OHTrp, 5-hydroxy-Trp; 5BrTrp, 5-bromo-Trp; 6BrTrp, 6-bromo-Trp; TrpRS, tryptophanyl-tRNA synthetase.

Nisin naturally lacks any Trp residue (Figure 1A). In this study, we used the nisin(I1W) mutant (Figure 1A) and evaluated the incorporation efficiency of several Trp analogues (Figure 1C) using the three different systems presented in Figure 2A. Previous studies indicated this mutant, compared to wild-type nisin, shows an improved antimicrobial activity against *L. lactis* NZ9000, while it is somewhat less active against *L. lactis* MG1363.^16,17,30^ To confirm overexpression of TrpRS in the pCZ and pNZ expression systems (Figure 2A1, A3), we performed SDS-PAGE gel analysis of harvested cells and observed a band at approximately 38 kDa (Figure S2), matching the theoretical mass of TrpRS. In contrast, the band was not present using the pNZ system without the TrpRS gene (Figures 2A2 and S2). These results demonstrate the overexpression of TrpRS in *L. lactis*, and the concurrent expression of TrpRS and nisin variant is expected to increase the yield and efficiency of Trp analogue incorporation.^27^ Using the pCZ system, mutant nisin(I1W) was well expressed when Trp or 5-hydroxyTrp (5OHTrp) was present in the growth medium (Figure 3A), which is consistent with previous findings.^18^ However, no nisin bands are visible when expressed in the presence of 5BrTrp or 6-bromtryptophan (6BrTrp). Because of the fairly high protein threshold concentration needed for protein precipitation using trichloroacetic acid (TCA) (>5 μg/mL), the supernatant was 10-fold concentrated before performing the TCA precipitation. Weak bands were observed when analyzed by tricine-SDS-PAGE gel analysis (Figure 3B). This result suggests that the BrTrp analogues can be incorporated using the pCZ-nisin system, albeit at a very low yield. With the pNZ systems, we were able to efficiently produce nisin when Trp or one of the Trp analogues (5OHTrp, 5BrTrp, or 6BrTrp) was present in the medium. Figure 3A illustrates the expression levels of nisin cultured in the presence of Trp, 5OHTrp, 5BrTrp, or 6BrTrp using the pNZ-nisin system without or with the TrpRS overexpression. Nisin was expressed at high levels when Trp was added to the medium, and notably, a comparable yield of 5OHTrp-, 5BrTrp-, or 6BrTrp-labeled nisin(I1W) was obtained. Together, using the zinc promoter for NisBTC expression and the nisin promoter for nisin results in a high expression of nisin(I1W), labeled with Trp or a Trp analogue.

### The Variants Were Correctly Modified and the Trp Analogues Were Efficiently Incorporated

**Figure 4:**
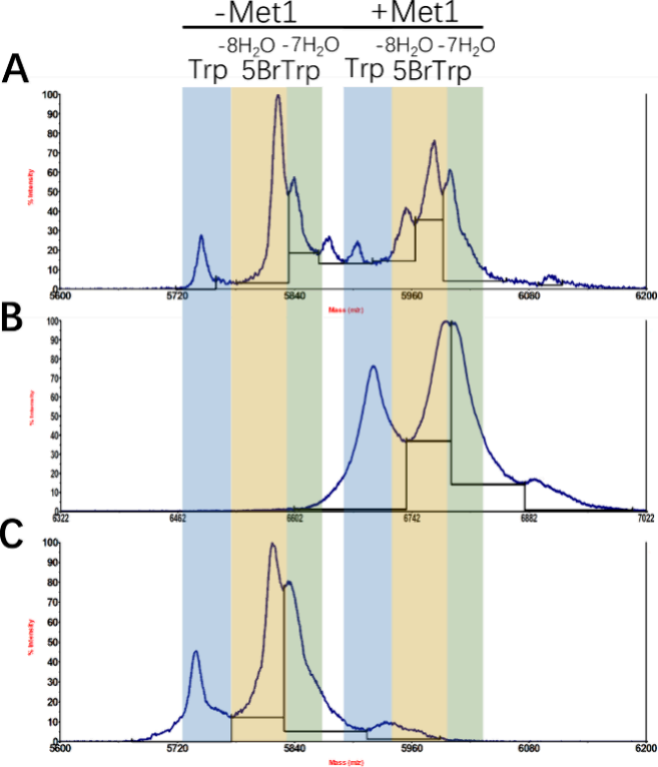
MALDI-TOF MS analysis of nisin labeled with 5BrTrp using the three cross-expression systems. (A) pCZ-nisin(I1W)-TrpRS system. (B) pNZ-nisin(I1W) system. In this system, Met1 is flanked by a 6-His tag. (C) pNZ-nisin(I1W)-TrpRS system. −8H_2_O, 8 times dehydration (fully dehydrated nisin). −7H_2_O, 7 times dehydration; Met1, the Met at residue position 1 in the leader part; usually, it has been cut off. Blue area, nisin labeled with Trp.

All samples were analyzed by MALDI-TOF MS to assess the efficiency of Trp analogue incorporation and the presence of post-translational modifications. In the leader part of prenisin, the first Met residue (Met1) is usually removed by the methionine aminopeptidase.^31^ Peptides produced by the pCZ-nisin system showed that half of them contained Met1, which is consistent with previous results (Figure 4A).^18,32^ For the pNZ-nisin-TrpRS system, the peptides without Met1 were dominant (Figure 4C, Table S1), as we found using this system for Met analogue incorporation.^29^ The reason might be the slower production rate or the prolonged presence in the bacterial cytoplasm. Met1 is present in isolated nisin when expressed by using the pNZ-nisin(I1W) system (Figure 4B). In this system, Met1 is next to a 6-His tag (Table S2), and this apparently prevents the methionine aminopeptidase from removing Met1.

The incorporation efficiency represents the ratio between the amounts of peptides containing the analogues and the total amount of peptides. In the pCZ-nisin system, the incorporation efficiency varied depending on the analogue used. The analogue 5OHTrp showed high incorporation efficiency (>99%), consistent with previous findings.^18^ The incorporation efficiencies for the two BrTrp analogues were 85% (5BrTrp) and 73% (6BrTrp), respectively (Table 1). We note that the yields of the latter two peptides were very low (Figure 3B). In the pNZ-system without overexpression of TrpRS, 5OHTrp consistently showed high incorporation efficiency (>99%), while the BrTrp incorporation efficiency was significantly lower (48–72%). However, with the TrpRS overexpression, the incorporation efficiency for BrTrp was greatly improved (≥80%), highlighting the importance of TrpRS coexpression. Petrović also reported a similar result, as 5-methyl-Trp incorporation efficiency increased when TrpRS was coexpressed.^27^

**Table 1:** Incorporation Efficiency of Trp Analogue Incorporation Using Different Cross-Expression Systems

system	analogue	incorporation efficiency (%)^a^
pCZ-nisin(I1W)-TrpRS	5OHTrp	>99%^a^
	5BrTrp	85%
	6BrTrp	73%
pNZ-nisin(I1W)	5OHTrp	>99%^a^
	5BrTrp	72%
	6BrTrp	48%
pNZ-nisin(I1W)-TrpRS	5OHTrp	>99%^a^
	5BrTrp	80%
	6BrTrp	83%

a >99% means the peak of the Trp-containing peptide was undetectable.

We observed that Trp analogue incorporation at position 1 had a negative influence on the dehydration of Thr, as 7 times dehydrated peptides were also observed when analogues were incorporated (Table S1, Figure 4). Particularly when BrTrp was incorporated, 40–50% of peptides were 7 times dehydrated (Figure 4). This may be attributed to the influence of the Thr2 residue. Previous studies have shown that substituting Ile1 in nisin with Trp results in the generation of two variants: the nisin(I1W) mutant as the primary product and a minor fraction of I1W/Dhb2T, wherein the Thr residue evades dehydration.^16^ The primary peak in the MALDI-TOF MS spectrum exhibited a mass of 5840 Da, aligning with that of fully cyclized nisin(I1W) after BrTrp incorporation (Figure S3B,C). Notably, there was no mass shift of +125 Da following the *N*-ethylmaleimide (NEM) alkylation assay (Figure S3A), which indicates the absence of free Cys in the BrTrp variants. The results of the NEM alkylation assay confirmed that the expressed peptides undergo complete cyclization facilitated by NisC. In summary, these results affirm the successful expression and postmodification of nisin variants labeled with a Trp analogue using the nisin-controlled gene expression (NICE) system^33^ combined with the NisBTC modification machinery.

Halogenation, particularly bromination, is a well-known post-translational Trp modification in RiPPs. In most cases where the site of substitution has been investigated, bromination has been observed to occur specifically at position 6 of the indole ring.^34^ These halogenated peptides are commonly found in marine organisms, likely due to the abundance of bromide ions in seawater (0.9 mM) and the expression of (halo)peroxidases that catalyze the bromination reaction.^24^ The *L. lactis* expression system makes it possible to produce BrTrp-labeled peptides within 24 h, while a halogenase-enzyme-based procedure can take up to 5 days.^25^ Together, in this study, we present a system for efficient incorporation of different BrTrp analogues (e.g., 5BrTrp and 6BrTrp) with high yield and incorporation efficiency.

### Optimization of the Expression for BrTrp Incorporation

**Figure 5:**
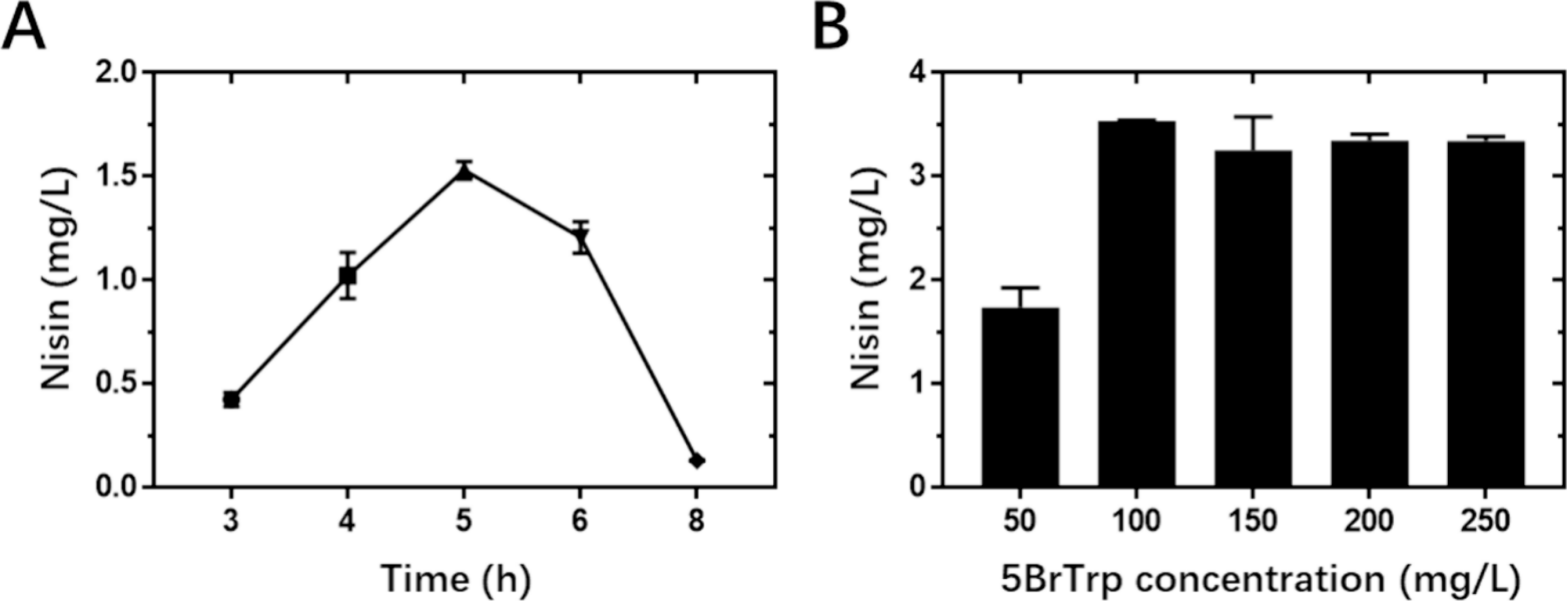
Optimization of the expression of nisin labeled with 5BrTrp. (A) The effect of the induction time, that is the time used to first induce nisin modification machinery NisBTC, on the peptide expression following supplementing the medium with 250 mg/L 5BrTrp. (B) The effect of the 5BrTrp concentration on the peptide expression using 5 h induction for NisBTC.

The influence of the analogue concentration and induction time on the expression of 5BrTrp incorporation was further evaluated (Figure 5, Figure S4). Figure 5A demonstrates the effect of induction time, defined as the time for inducing the NisBTC modification enzymes before the addition of the analogue and inducing nisin expression. The amount of nisin increased when the induction time was prolonged from 3 h to 5 h. A further increase of the time from 5 h to 8 h led to a decrease in the product formation. As shown in Figure 5B, the Trp analogue 5BrTrp concentration significantly affected peptide formation. The yield gradually increased from 50 mg/L to 100 mg/L of 5BrTrp, but did not increase further beyond 100 mg/L. Therefore, the 5BrTrp concentration adopted was 100 mg/L for the next experiments. A 36 mg/L Trp concentration is typically used for normal protein expression in the chemically defined media.^35^ The need for a higher analogue concentration may reflect the difficulty of charging BrTrp to tRNA^Trp^ by tryptophanyl-tRNA synthetase. Together, the optimal conditions for BrTrp incorporation are a final BrTrp concentration of 100 mg/L and a 5 h induction time, at which up to 3.5 mg/L pure peptide can be isolated.

### Antimicrobial Activity of the Nisin Variants

The nisin variants were produced (Figures S5 and S6) at the milligram scale for antimicrobial activity tests. We determined the minimum inhibitory concentration (MIC) values for *L. lactis* and five Gram-positive pathogenic strains, including two *Staphylococci*, two *Enterococci*, and one *Bacillus cereus* (Table 2). The results showed that changing Ile to Trp at position 1 of nisin only decreased the activity against *L. lactis* MG1363 (2-fold), but did not affect the activity against the other tested strains. These results are consistent with previous findings.^17^ Incorporating 5OHTrp led to a 2-fold decrease in activity against *L. lactis*, *S. aureus*, and *E. faecium*, suggesting that the polarity or H-bond forming capability of the Trp1 side chain affects the antimicrobial activity. Incorporating 6BrTrp led to a 2-fold decrease in activity against *L. lactis*, one *S. aureus*, and *E. faecalis*, with no change in activity against the other two strains. Labeling with 5BrTrp retained activity against *L. lactis*, one *S. aureus* strain, and *E. faecalis*, but interestingly showed a higher activity against two other strains, namely, *S. aureus* LMG15975 (MRSA) and *B. cereus* CH-85, making it more active than wild-type nisin against these two strains. Our understanding of why the antimicrobial activity of a RiPP varies for different microorganisms is in its infancy, and this is also true for one of the best studied RiPPs, nisin. The results for the nisin(I1W) variant labeled with either 5BrTrp or 6BrTrp demonstrate that a small structural change in the Trp1 side chain has quite an impact on its antimicrobial activity (Table 2). In summary, in this study, we compared the antibacterial activity of wild-type nisin with nisin mutant I1W and Trp analogue labeled variants of this mutant against six pathogens. For most nisin variants, a similar or lower activity was observed compared to that of wild-type nisin. But the 5BrTrp-labeled nisin variant exhibited somewhat improved activity against two tested human pathogens, including a WHO priority pathogen.

**Table 2:** Antimicrobial Activity of Nisin (μg/mL) and its Derivatives against Microorganisms^a^

	MIC (μg/mL)				
		nisin I1W			
organism and type	nisin WT	Trp	5OHTrp	5BrTrp	6BrTrp
*Lactococcus lactis* MG1363	0.02	0.04	0.08	0.04	0.08
*Staphylococcus aureus* LMG15975 (MRSA)^a^	6.25	6.25	>12.5	3.13^b^	6.25
*Staphylococcus aureus* LMG10147	6.25	6.25	12.5	6.25	12.5
*Enterococcus faecalis* LMG16216 (VRE)^a^	12.50	12.50	12.5	12.50	25
*Bacillus cereus* CH-85	6.25	6.25	6.25	3.13^b^	6.25
*Enterococcus faecium* LMG16003 (VRE)^a^	3.13	3.13	6.25	3.13	3.13

a VRE, vancomycin-resistant *Enterococci*; MRSA, methicillin-resistant *Staphylococcus aureus*.

b The modified nisin variant shows improved antibacterial activity compared to wild-type (WT) nisin.

### 5-Brominated Trp1 Nisin Variant Binds to Lipid II and Shows Slower Bactericidal Activity than Nisin

**Figure 6:**
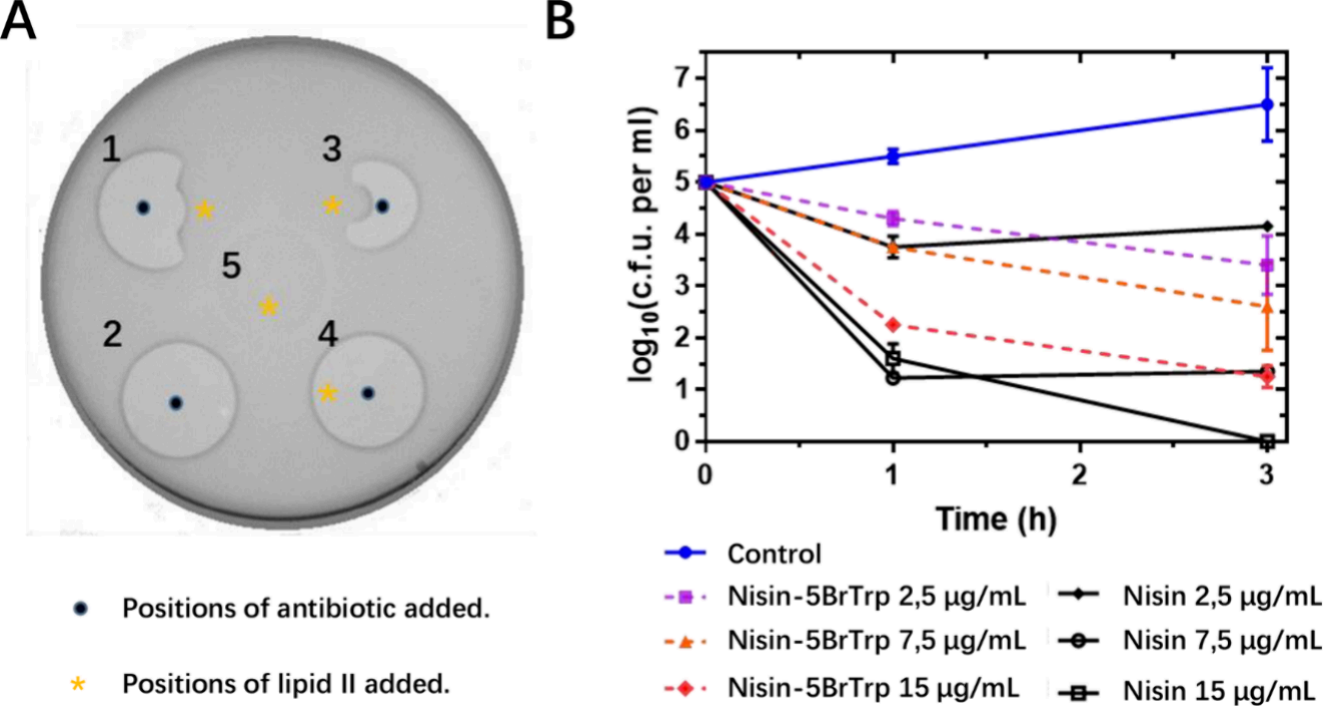
Preliminary results of the mode of action of the brominated nisin variant against *B. cereus*. (A) Spot-on-lawn assay to test the lipid II binding activity. 1, wild-type nisin; 2, wild-type nisin; 3, 5BrTrp-labeled nisin(I1W) variant; 4, daptomycin; and 5, H_2_O. *, the position lipid II was added (600 μM, 2 μL). (B) Viability of an exponential culture of *B. cereus* exposed to lantibiotics at the indicated concentrations.

Nisin, a bactericidal lantibiotic, is well-known for its ability to inhibit cell wall biosynthesis by binding lipid II and form pores in target cell membranes.^5^ To investigate whether the brominated modification affects the mode of action of nisin, we first assessed its binding ability to lipid II (Figure 6A). We observed that externally added purified lipid II decreased the antimicrobial activity of nisin and the 5BrTrp nisin(I1W) variant against *B. cereus*, resulting in a disruption of the normally circular antibiotic-induced halo. We used daptomycin as a non-lipid II-binding antibiotic, which maintained its antimicrobial activity against *B. cereus* after the addition of purified lipid II, resulting in a circular halo. It demonstrates that despite its structural modification, the brominated nisin variant retains its ability to bind to lipid II, as does nisin. To investigate the potential impact of brominated modification on nisin’s bactericidal activity, we evaluated the killing activity of nisin and the brominated nisin variant against *B. cereus*, as shown in Figure 6B. At the lowest concentration tested (2.5 μg/mL), the two peptides showed only slight killing within 3 h. Higher lantibiotic concentrations resulted in stronger killing for both wild-type nisin and the brominated nisin variant. At a concentration of 15 μg/mL, nisin completely killed all cells within 3 h. However, the same concentration of the brominated nisin variant showed slower bactericidal activity than did nisin. These results suggest that the presence of an additional bromine atom decreases somewhat the pore-forming ability of nisin(I1W).

## Conclusions

This study represents a significant advancement in the efficient incorporation of BrTrp into RiPPs as exemplified by nisin engineering. For this, an *L. lactis* Trp auxotroph-based cross-expression system was utilized to produce RiPPs in high yield. This study demonstrates the potential to incorporate Trp analogues in lanthipeptides, resulting in new-to-nature antimicrobial products, in this way, extending the toolbox for (lanthi)peptide drug improvement and discovery.

## Experimental Section

### General Experimental Procedures

All reagents for molecular biology experiments were obtained from Thermo Fisher Scientific (Waltham, MA, USA) unless otherwise stated, and all other chemicals were acquired from Sigma-Aldrich (St. Louis, MO, USA). The Trp analogue 6-bromo-dl-Trp was obtained from Biosynth Carbosynth (Lelystad, The Netherlands), while 5-bromo-dl-Trp and 5-hydroxy-l-Trp were purchased from Sigma-Aldrich. Lipid II was synthesized and purified as described in a previous study and was kindly provided by Prof. Dr. E. J. (Eefjan) Breukink.^37^

### Bacterial Strains, Plasmids, and Growth Conditions

The bacterial strains and plasmids used in this study are given in Table S2. *L. lactis* strains were grown in GM17 (M17 broth supplemented with 0.5% glucose) at 30 °C and supplemented with 5 μg/mL chloramphenicol and/or erythromycin when appropriate. Cloning and plasmid maintenance were performed in *L. lactis* NZ9000, while peptide expression was performed in *L. lactis* PA1002. Protein expression and incorporation of Trp analogues were carried out in chemically defined medium lacking tryptone (CDM-P).^35^

### Molecular Biology Techniques

The PCR primers used in this study are listed in Table S3 and were purchased from Biolegio B.V. (Nijmegen, The Netherlands). Mutant plasmids were constructed as previously described.^36^ For plasmid pNZ-nisin(I1W)-TrpRS, the purified PCR products were fused using the Gibson assembly master mix (BIOKE, Leiden, The Netherlands). The plasmid sequence was verified by sequencing.

### Expression of ncAA-Incorporated Nisin

To explore the incorporation of ncAA, we performed small-scale (20 mL) expression and purification experiments. For the pCZ-nisin expression system, we followed a previously described procedure for the precipitation of the precursor peptide.^18^ For the pNZ-nisin system, *L. lactis* PA1002 cells harboring the *nisBTC* plasmid were electroporated with a plasmid carrying the *nisA* gene (100 ng) and grown overnight on GM17 agar plates supplemented with chloramphenicol (5 μg/mL) and erythromycin (5 μg/mL) at 30 °C. A single colony was picked and cultured in 4 mL of GM17CmEm medium until an OD_600_ value of approximately 0.4 was reached. Next, 0.5 mM ZnSO_4_ was added to induce the expression of the nisin modification machinery NisBTC. After 3 h, the cells were washed three times with phosphate-buffered saline (PBS, pH 7.2) and resuspended in 20 mL of Trp-free CDM-P.^35^ Following a 1 h starvation period, Trp or Trp analogue (250 mg/L) and 8 ng/mL nisin were added to induce peptide expression. After overnight growth, the supernatant was collected by centrifugation at 8000*g* for 15 min, and nisin was precipitated with 10% TCA on ice for at least 2 h. The precipitate was then centrifuged at 10000*g* and 4 °C for 45 min, washed with 10 mL of ice-cold acetone to remove TCA, and dried in the fume hood or resuspended in 0.2 mL of 0.05% aqueous acetic acid solution for further analysis.

### Tricine-SDS-PAGE Analysis

The precipitated peptides were analyzed using the Tricine-SDS-PAGE gel system described by Schägger.^39^ Briefly, 10 μL of the sample was mixed with 8 μL of loading dye and loaded onto a 16% gel. The proteins were visualized by staining with Coomassie brilliant blue G-250.

### Mass Spectrometry Analysis

To analyze the mass of peptides or analogue incorporation efficiency, 1 μL of the peptide was spotted onto a target and washed several times with Milli-Q water. An equal volume of matrix solution (5 mg/mL α-cyano-4-hydroxycinnamic acid dissolved in 50% acetonitrile containing 0.1% trifluoroacetic acid) was then applied to the sample. Mass spectra (MS) were obtained by using an Applied Biosystems 4800 Plus matrix-assisted laser desorption/ionization time-of-flight analyzer (MALDI-TOF) operating in linear mode with external calibration. The analogue incorporation efficiency was calculated by comparing the peak areas of analogue-containing peptides to those of Trp-containing peptides.

Liquid chromatography–tandem mass spectrometry (LC-MS/MS) was conducted to explore the position of the analogue incorporation. LC-MS analysis utilized an Ultimate 3000 UPLC system coupled with a Q-Exactive mass spectrometer, employing an ACQUITY BEH C_18_ column (2.1 × 50 mm, 1.7 μm particle size, 200 Å; Waters), an HESI ion source, and an Orbitrap detector. Each run involved injecting a 10 μL sample, which was then separated using a gradient of MeCN containing 0.1% formic acid (v/v) at a flow rate of 0.5 mL/min. MS/MS data were acquired separately in PRM mode, targeting the doubly or triply charged ions of the compound of interest.

### Optimization of the Expression of Nisin Labeled with BrTrp

To maximize nisin production, we investigated the optimal induction time and concentration of the Trp analogue 5BrTrp. Specifically, the impact of induction time (ranging from 3 to 8 h) on nisin production was studied by adding 250 mg/L 5BrTrp to the medium after *nisBTC* induction. The effect of different concentrations of 5BrTrp (ranging from 50 to 250 mg/L) on nisin production was evaluated by adding it to the medium after 5 h of *nisBTC* induction.

### Purification and Quantification of Nisin Variants with Trp or Trp Analogues

To obtain larger amounts of nisin variants, we conducted experiments on a 2 L scale. The supernatant was adjusted to pH 7.0 and incubated with purified NisP^40,41^ at 37 °C for 3–6 h to cleave off the leader sequence. Subsequently, the supernatant was loaded onto a C_18_ open column (Spherical C_18_, 20 g, particle size: 40–75 μm, Sigma-Aldrich). The column was washed with different concentrations (30%, 35%, 40%, and 60%) of buffer B (buffer A, distilled water with 0.1% trifluoroacetic acid; buffer B, acetonitrile with 0.1% trifluoroacetic acid) using 40 mL of each concentration. The active fractions were lyophilized and further purified using an Agilent 1200 series high-performance liquid chromatograph (HPLC) equipped with a C_12_ column (Jupiter 4 μm, Proteo 90 Å, 250 × 4.6 mm, Phenomenex). We collected the peak with activity and the correct molecular mass, lyophilized it, and stored it at 4 °C until further use.

The nisin concentration was measured using HPLC following the protocol described by Schmitt et al.^35^ The amount of nisin variants containing Trp or its analogues were determined using a NanoDrop spectrophotometer (Thermo Scientific) calibrated with the extinction coefficient predicted by ExPASy (http://web.expasy.org/protparam/).

### Minimal Inhibitory Concentration (MIC) Assay

MIC values were determined using broth microdilution according to standard guidelines.^42^ The inoculum was adjusted to approximately 5 × 10^5^ CFU/mL, and the MIC was defined as the lowest concentration of antimicrobial compound with no visible growth after overnight incubation at 37 °C (or at 30 °C for *L. lactis* strain).

### Spot-on-Lawn Assay to Test Lipid II Binding Assay

A 0.1% (v/v) inoculum of *B. cereus* CH-85 from an overnight culture was added to 1.5% (w/v) LB agar at 45 °C, and 10 mL of the mixture was poured onto a plate. The binding of the peptide to lipid II was evaluated by spotting purified lipid II (0.8 mol/L, 2 μL)^38,41^ at the edge of the 0.2 mg antibiotic inhibition halo. Specifically, 5 μL of the antibiotic was loaded onto the agar plate, and after drying, the lipid II solution was spotted at the edge of the inhibition halo. The plate was then incubated overnight at 37 °C.

### Time–Kill Assay

The antimicrobial activity of nisin and its brominated variants was evaluated against *B. cereus* CH-85 using a previously described procedure.^38,41^ Briefly, an overnight culture of *B. cereus* was diluted 50-fold in LB medium and grown at 37 °C with aeration until reaching an OD_600_ of 0.5, after which the cell concentration was adjusted to 5 × 10^5^ cells per mL. Bacteria were then exposed to different concentrations of nisin or brominated nisin variant in culture tubes at 37 °C and 180 rpm, with untreated bacteria serving as a negative control. At desired time points, 50 μL aliquots were taken, and 10-fold serial dilutions were plated on LB agar plates. After overnight incubation at 37 °C, colonies were counted, and colony-forming units (CFU) per milliliter were calculated. Each experiment was performed in triplicate.

## Data Availability

All data supporting the findings of this study are available within the paper and its Supporting Information.

## Supporting Information Available

Nisin variant production and Trp analogue incorporation systems; expression of TrpRS recombinant protein in *L. lactis* PA1002, visualized via SDS-PAGE gel; *N*-ethylmaleimide alkylation assay to determine the level of cyclization; Coomassie-blue-stained tricine-SDS-PAGE gel analysis of optimization of the expression of nisin labeled with 5BrTrp; MALDI-TOF MS analysis of HPLC-purified peptides; LC-MS/MS spectrum of nisin(I1W) labeled with 5BrTrp; a table with MS data of the nisin variants; a table summarizing the plasmids and strains used in this study; and a table summarizing the primers used in this study (PDF)
