# Supplementary material for: Facile Halogenation of Antimicrobial Peptides As Demonstrated by Producing Bromotryptophan-Labeled Nisin Variants with Enhanced Antimicrobial Activity
Source: J Nat Prod. 2024 Jun 18;87(6):1548–55. doi: 10.1021/acs.jnatprod.4c00118 (PMC11217935; doi:10.1021/acs.jnatprod.4c00118)
Supplement: Supplementary file 1 — np4c00118_si_001.pdf [file np4c00118_si_001.pdf]

Facile Halogenation of Antimicrobial Peptides as Demonstrated by Producing Bromotryptophan-Labeled Nisin Variants with Enhanced Antimicrobial Activity

Longcheng Guo [a], Oscar P. Kuipers [a], Jaap Broos [a,*]

[a] Department of Molecular Genetics, Groningen Biomolecular Sciences and Biotechnology Institute, University of Groningen, Groningen, the Netherlands

* Correspondence to Jaap Broos, j.broos@rug.nl.

SUPPORTING INFORMATION

Table of Contents

Figure S1. Nisin variants production and Trp analogue incorporation systems……………………….…..…3

Figure S2. Expression of TrpRS recombinant protein in *L. lactis* PA1002, visualized via SDS-PAGE gel……………………………………….…………….…………….………...……4

Figure S3. N-ethylmaleimide (NEM) alkylation assay to determine the level of cyclization…….……….5-6

Figure S4. Coomassie-blue stained tricine-SDS-PAGE gel analysis for the optimization of the expression of nisin labeled with 5BrTrp…………………………….…………….…………….………….……...……7

Figure S5. MS analysis of HPLC purified nisin variants with Trp or analogue incorporated……………..8

Figure S6. High resolution MS spectra of nisin(I1W) labelled with 5BrTrp…………………………...…..9

Table S1. The molecular masses of nisin(I1W) after Trp analogues are incorporated using the three different incorporation systems……………………….…………….……………..…………………….……..10

Table S2. Bacterial strains and plasmids used in this study…….…………………………..…………...11

Table S3. Primers used in this study…………………………………………….……….……….………...12

References…………….……………….…………………….………..……………….……………….………13

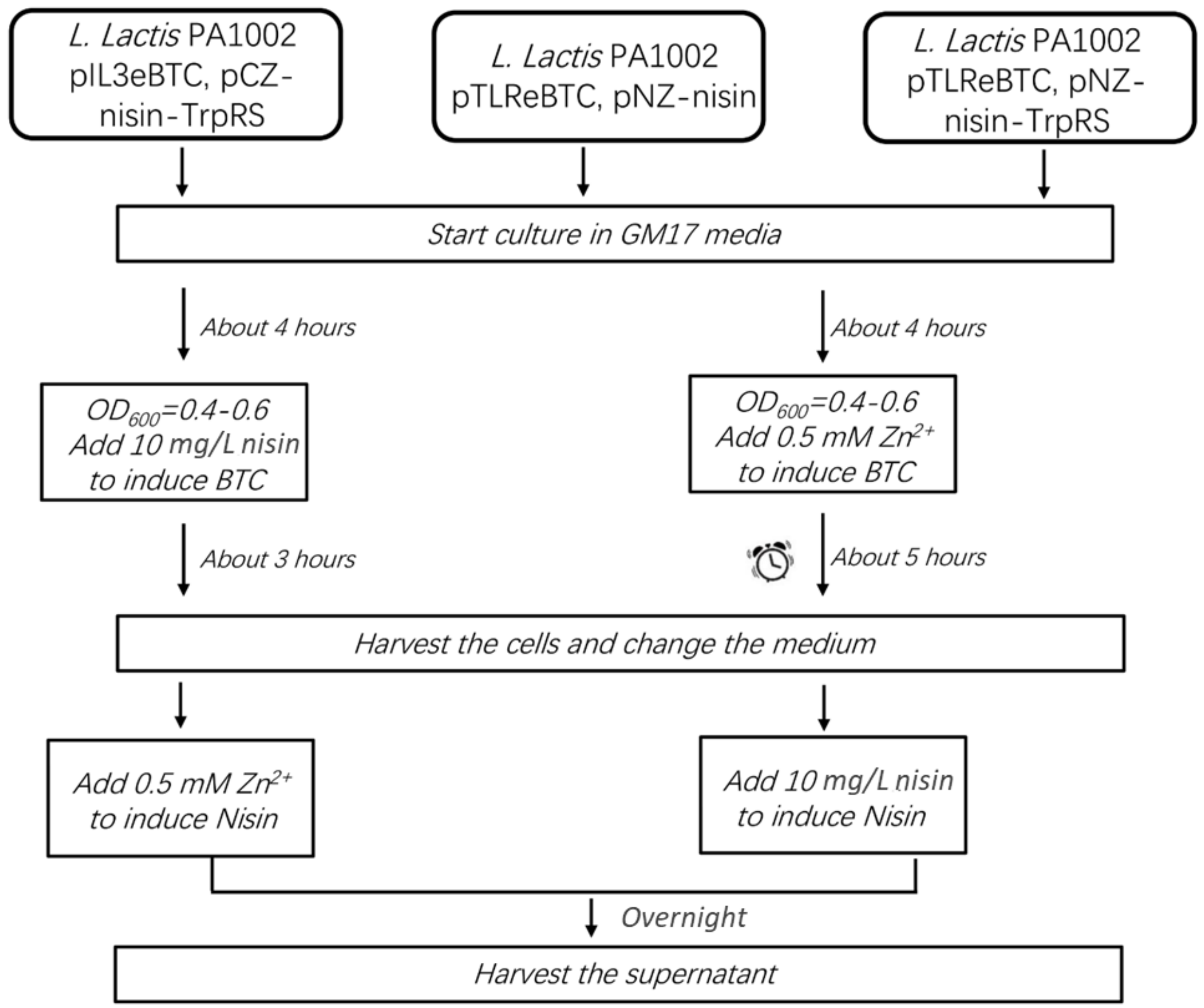

Figure S1. Nisin variants production and Trp analogue incorporation systems.

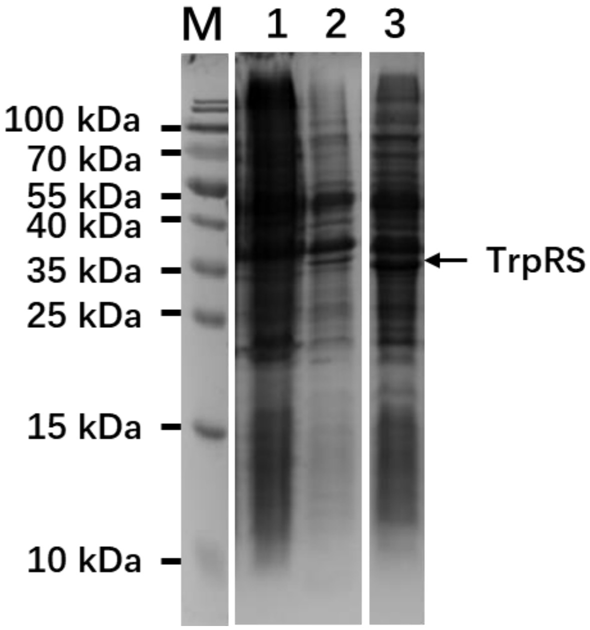

Figure S2. Expression of TrpRS recombinant protein in *L. lactis* PA1002, visualized via SDS-PAGE gel. The sample was prepared as whole cell extracts of *L. lacti*s PA1002 harboring the TrpRS (lane **2, 3**) or without the expression of TrpRS (lane **1**). Black arrow indicates TrpRS band. Lane **M**, protein maker; Lane **1**, pNZ-nisin(I1W); Lane **2**, pNZ-nisin(I1W)-TrpRS; Lane **3**, pCZ-nisin(I1W)-TrpRS.

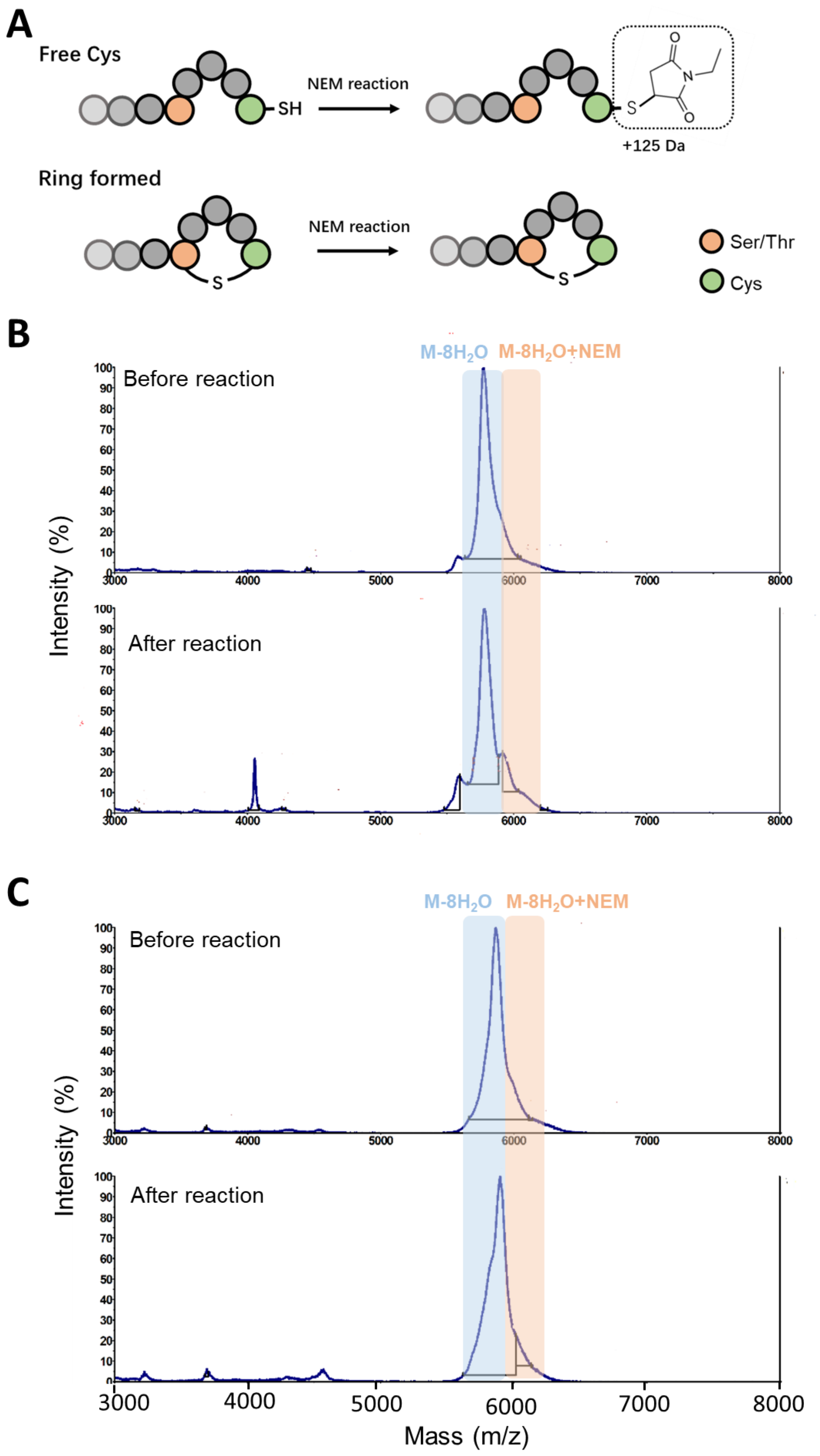
A
Free Cys
SH
NEM reaction
S
O
N
O
+125 Da
Ring formed
S
NEM reaction
S
Ser/Thr
Cys
B
M-8H2O
M-8H2O+NEM
Before reaction
After reaction
Intensity (%)
C
M-8H2O
M-8H2O+NEM
Before reaction
After reaction
Intensity (%)
Mass (m/z)

Figure S3. N-ethylmaleimide (NEM) alkylation assay to determine the level of cyclization. (**A**) A schematic illustration for the reaction of NEM with free Cys, resulting in a 125 Da increase. Conversely, when Cys forms a ring with dehydrated amino acids, no additional molecular mass is observed. (**B**) The mass of the major peak after NEM addition (bottom) aligns with the mass of nisin(I1W) labeled with 5BrTrp before NEM addition (top), confirming the absence of free Cys in the majority of the peptide. (**C**) Similar findings were observed for the nisin with 6BrTrp incorporation, where the mass after NEM addition (bottom) matches the mass of the nisin with 6BrTrp incorporation before NEM addition (top).

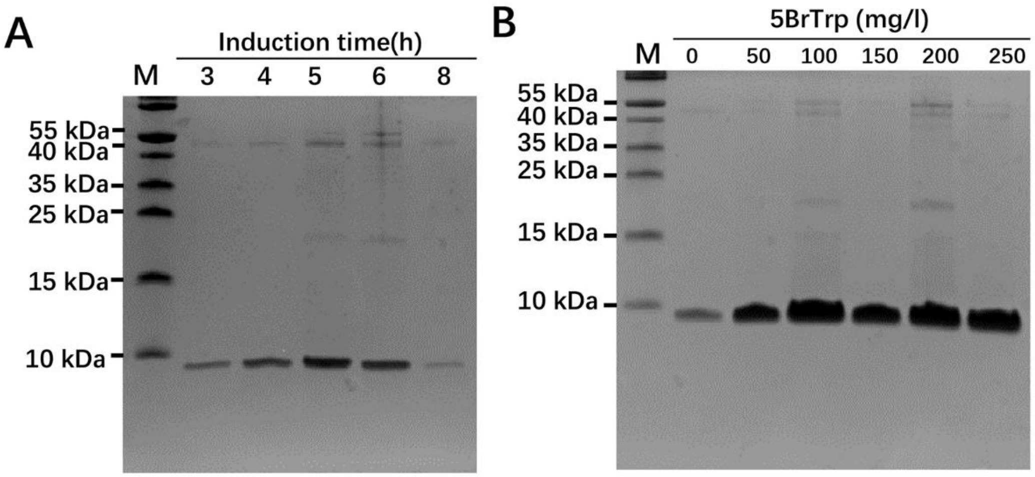

Figure S4. Coomassie-blue stained tricine-SDS-PAGE gel analysis for the optimization of the expression of nisin labeled with 5BrTrp. (**A**) The effect of the induction time, that is the time used to first induce nisin modification machinery NisBTC, on the peptide expression following 250 mg/L 5BrTrp supplemented. (**B**) The effect of the 5BrTrp concentration on the peptide expression using 5 hours' induction of NisBTC before.

A

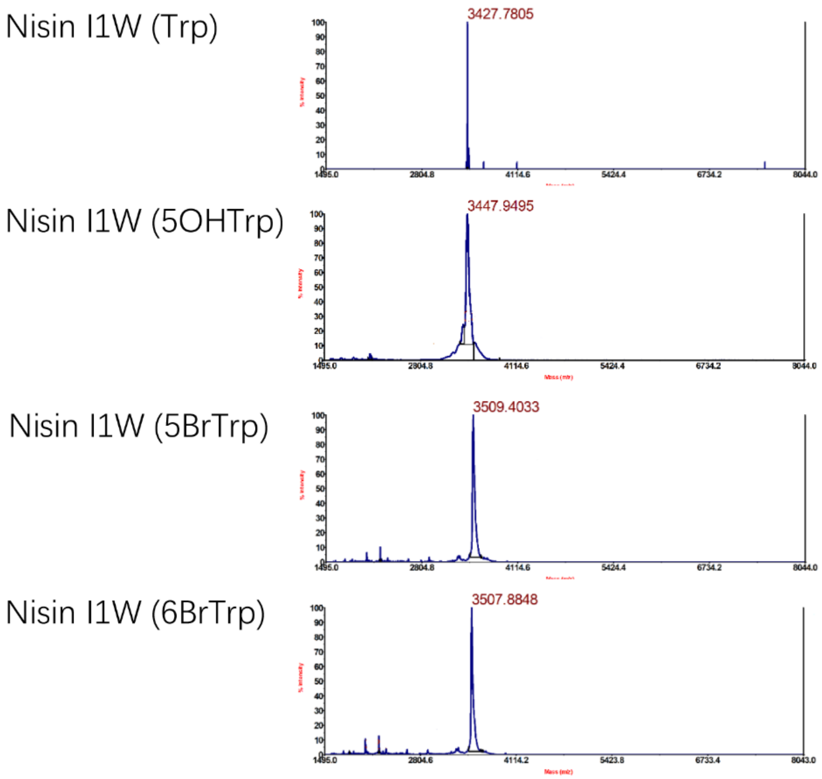

B

Mutation	Incorporation	Modification	Mass (Da) Predicated	Mass (Da) Observed	Mass (Da) Difference
Nisin I1W	Trp	$-8H_2O$	3427.24	3427.78	<1
	5OHTrp	$-8H_2O$	3443.23	3447.95	4.72
	5BrTrp	$-8H_2O$	3506.13	3509.40	3.27
	6BrTrp	$-8H_2O$	3506.13	3507.88	1.75

Figure S5. MS analysis of HPLC purified nisin variants with Trp or analogue incorporated. (**A**) MALDI-TOF MS analysis. (**B**) Predicted and observed mass of nisin variants.

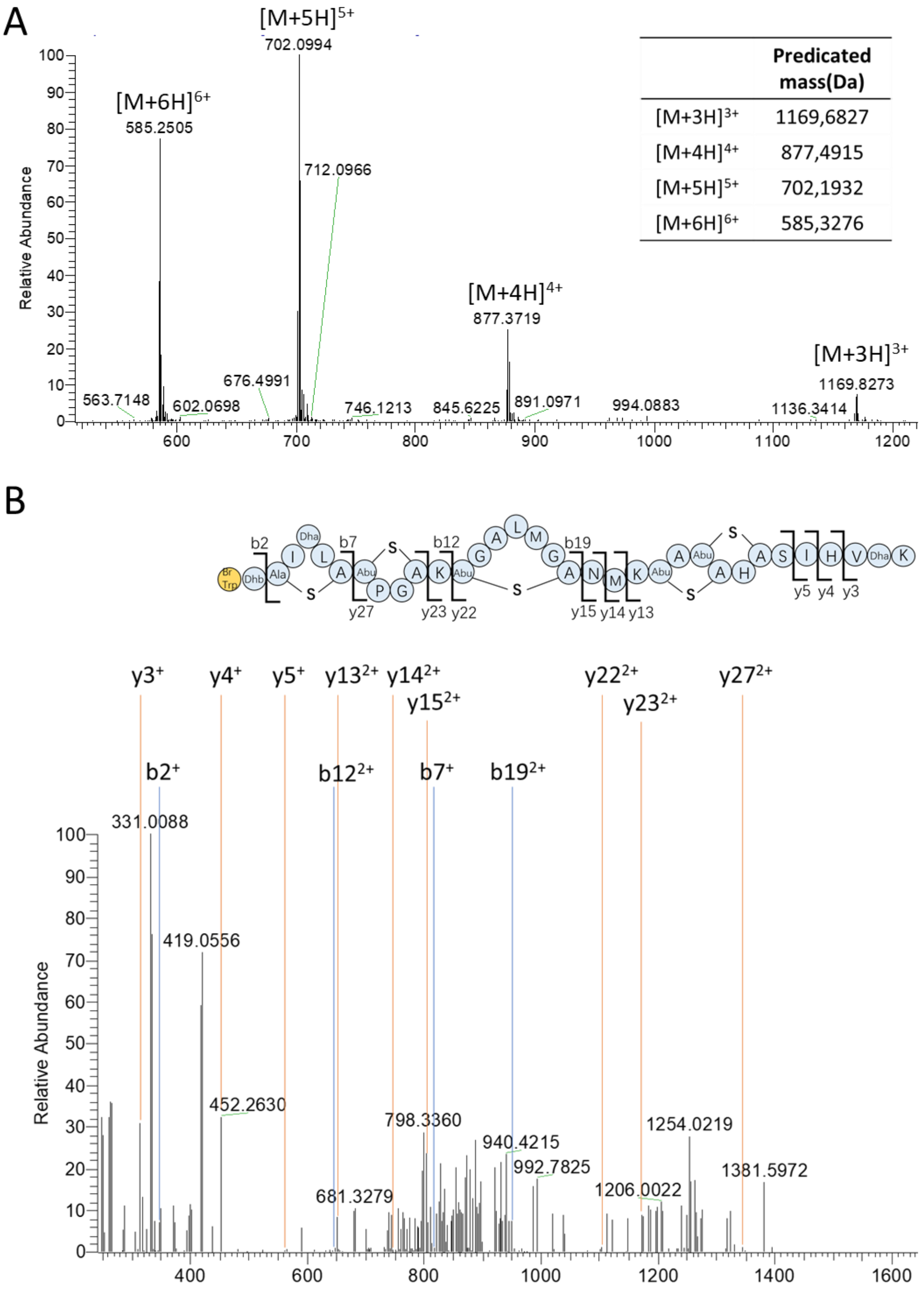

Figure S6. (**A**) High resolution MS spectra of nisin(I1W) labelled with 5BrTrp. (**B**) LC-MS/MS spectrum of nisin(I1W) labelled with 5BrTrp. The detected mass of fragment b2 (BrTrp-Dhb) was 366.21 Da (predicted 366.10 Da) confirming that 5BrTrp is incorporated at residue position 1.

Table S1. The Molecular Masses of Nisin(I1W) after Trp Analogues are Incorporated using the Three Different Incorporation Systems.

Analog	Modification	Predicted mass (Da)		Measured mass (Da)							
				Trp		5OHTrp		5BrTrp		6BrTrp	
		+Met1	-Met1	+Met1	-Met1	+Met1	-Met1	+Met1	-Met1	+Met1	-Met1
**pCZ-nisin(I1W)-TrpRS**											
Trp	$-8H_2O$	5892.03	5760.82	5894.75	5762.89						
Trp	$-8H_2O$+CN	5917.03	5785.82	5918.30							
5OHTrp	$-8H_2O$	5908.02	5776.81				5773.02				
5OHTrp	$-7H_2O$	5926.02	5794.81			5934.55					
5BrTrp	$-8H_2O$	5970.92	5839.71		5765.04			5976.07	5843.46		
5BrTrp	$-8H_2O$+CN	5995.92	5864.71	5923.83				6002.05			
5BrTrp	$-7H_2O$	5988.92	5857.71						5859.32		
5BrTrp	$-7H_2O$+CN	6013.92	5882.71					6018.85			
6BrTrp	$-8H_2O$	5970.92	5839.71	5925.67	5766.63					5977.41	5846.87
6BrTrp	$-8H_2O$+CN	5995.92	5864.71							6006.66	
6BrTrp	$-7H_2O$	5988.92	5857.71								5861.98
6BrTrp	$-7H_2O$+CN	6013.92	5882.71							6022.95	
**pNZ-nisin(I1W)**											
Trp	$-8H_2O$	6714.88	6583.67	6716.67							
5OHTrp	$-8H_2O$	6730.87	6599.66			6729.15					
5OHTrp	$-7H_2O$	6748.87	6617.66			6744.12					
5BrTrp	$-8H_2O$	6793.77	6662.56	6717.37				6795.47			
5BrTrp	$-7H_2O$	6811.77	6680.56					6814.24			
6BrTrp	$-8H_2O$	6793.77	6662.56	6710.00				6662.16			
6BrTrp	$-7H_2O$	6811.77	6680.56					6680.45			
**pNZ-nisin(I1W)-TrpRS**											
Trp	$-8H_2O$	5892.03	5760.82	5889.88	5763.00						
5OHTrp	$-8H_2O$	5908.02	5776.81			5906.06	5777.22				
5OHTrp	$-7H_2O$	5926.02	5794.81				5795.74				
5BrTrp	$-8H_2O$	5970.92	5839.71		5759.35			5965.66	5840.14		
5BrTrp	$-7H_2O$	5988.92	5857.71						5856.30		
6BrTrp	$-8H_2O$	5970.92	5839.71		5759.23						5840.50
6BrTrp	$-7H_2O$	5988.92	5857.71								5855.91

+Met1, with N-terminal first position Met; −Met1, without first position Met; $-8H_2O$, $-7H_2O$, variant times dehydration; +CN, extra 25Da mass added due to CN from matrix.

Table S2. Bacterial Strains and Plasmids Used in this Study.

Strains or plasmids	Characteristics	Reference
Strain		
Lactococcus lactis NZ9000	Plasmid construction and maintenance	1
Lactococcus lactis PA1002	*L. lactis* Trp auxotroph, peptide expression	2
Lactococcus lactis MG1363	indicator strain	Lab collection
Bacillus cereus CH-85	indicator strain	Lab collection
Staphylococcus aureus LMG10147	indicator strain	Lab collection
Staphylococcus aureus LMG15975	indicator strain, MRSA	Lab collection
Enterococcus faecium LMG16003	indicator strain, VRE	Lab collection
Enterococcus faecalis LMG16216	indicator strain, VRE	Lab collection
plasmid		
pIL3EryBTC	*nisBTC*, encoding nisin modification machinery, P_{nisA} promoter, Ery^R	3
pTLReBTC	*nisBTC*, encoding nisin modification machinery, P_{czcD} promoter, Ery^R	4
pNZ-nisA	*nisA*, encoding NisA, P_{nisA} promoter, Cm^R	3
pNZnisP8H	*nisP*, encoding NisP mutant, with 8 histidines, Cm^R	5
pCZ-nisin(I1W)-TrpRS	Nisin mutation (I1W) with the overexpression TrpRS, P_{czcD} promoter, Cm^R	This study
pNZ-nisin(I1W)	Nisin mutation (I1W) with 6 histidines in the leader, P_{nisA} promoter, Cm^R	6
pNZ-nisin(I1W)-TrpRS	Nisin mutation (I1W) with the overexpression TrpRS, P_{nisA} promoter, Cm^R	This study

Table S3. Primers Used in this Study.

Primer	Template	Nucleic acid sequences (5' to 3')	Characteristic
pNZ-nisin-TrpRS construction (Gibson method)			
pNZ-nisin_fwd	pNZ-nisA	TTTTAGGTAAGCTTTCTTTGAACCAAAATTAGAAAAC	
pNZ-nisin_rev		GTTTTGTCATTTATTTGCTTACGTGAATACTACAATG	
TrpRS_fwd	pCZ-nisin(I1W)-TrpRS	AAGCAAATAAATGACAAAACCAACTATTTTAACC	
TrpRS_rev		CAAAGAAAGCTTACCTAAAATAATTGATTCCCATTG	
Mutation (Self-ligation)			
I1W_fwd	pNZ-nisin-TrpRS	CCACGCTGGACAAGTATTTCGCTATGTACACCCG	
I1W_rev		TGATGCACCTGAATCTTTCTTCGAAAC	5'- phosphorylation

REFERENCES

1. Kuipers, O. P.; de Ruyter, P. G.; Kleerebezem, M.; de Vos, W. M. *Trends. Biotechnol.* **1997**, *15* (4), 135-140.

2. El Khattabi, M.; Van Roosmalen, M. L.; Jager, D.; Metselaar, H.; Permentier, H.; Leenhouts, K.; Broos, J. *J. Biochem.* **2008**, *409* (1), 193-198.

3. van Heel, A. J.; Mu, D.; Montalbán-López, M.; Hendriks, D.; Kuipers, O. P. *ACS Synth. Biol.* **2013**, *2* (7), 397-404.

4. Guo, L.; Wang, C.; Broos, J.; Kuipers, O. P. *J. Biol. Chem.* **2023**, *299* (7), 104845.

5. Montalbán-López, M.; Deng, J.; Van Heel, A. J.; Kuipers, O. P. *Front. Microbiol.* **2018**, *9*, 160.

6. Kuipers, O. P.; Rollema, H. S.; Beerthuyzen, M. M.; Siezen, R. J.; De Vos, W. M. *Int. Dairy J.* **1995**, *5* (8), 785-795.
